# Identification of Hypoxia Signature to Assess the Tumor Immune Microenvironment and Predict Prognosis in Patients with Ovarian Cancer

**DOI:** 10.1155/2021/4156187

**Published:** 2021-12-14

**Authors:** Chunyan Wei, Xiaoqing Liu, Qin Wang, Qipei Li, Min Xie

**Affiliations:** ^1^Department of Gynaecology and Obstetrics, The Second Affiliated Hospital of Xi'an Jiaotong University, Xi'an, China; ^2^Department of Gynaecology and Obstetrics, Maternal and Child Health Hospital of Shangzhou District, Shangluo, Shanxi Province, China

## Abstract

**Background:**

The 5-year overall survival rate of ovarian cancer (OC) patients is less than 40%. Hypoxia promotes the proliferation of OC cells and leads to the decline of cell immunity. It is crucial to find potential predictors or risk model related to OC prognosis. This study aimed at establishing the hypoxia-associated gene signature to assess tumor immune microenvironment and predicting the prognosis of OC.

**Methods:**

The gene expression data of 378 OC patients and 370 OC patients were downloaded from datasets. The hypoxia risk model was constructed to reflect the immune microenvironment in OC and predict prognosis.

**Results:**

8 genes (AKAP12, ALDOC, ANGPTL4, CITED2, ISG20, PPP1R15A, PRDX5, and TGFBI) were included in the hypoxic gene signature. Patients in the high hypoxia risk group showed worse survival. Hypoxia signature significantly related to clinical features and may serve as an independent prognostic factor for OC patients. 2 types of immune cells, plasmacytoid dendritic cell and regulatory T cell, showed a significant infiltration in the tissues of the high hypoxia risk group patients. Most of the immunosuppressive genes (such as ARG1, CD160, CD244, CXCL12, DNMT1, and HAVCR1) and immune checkpoints (such as CD80, CTLA4, and CD274) were upregulated in the high hypoxia risk group. Gene sets related to the high hypoxia risk group were associated with signaling pathways of cell cycle, MAPK, mTOR, PI3K-Akt, VEGF, and AMPK.

**Conclusion:**

The hypoxia risk model could serve as an independent prognostic indicator and reflect overall immune response intensity in the OC microenvironment.

## 1. Introduction

Ovarian cancer (OC) is characterized by relatively high incidence, high mortality rate, and poor prognosis [1, 2]. Poor differentiation of tumor, higher stage of disease, the presence of residual disease after cytoreductive surgery, older age, smoking, excessive weight, and lack of physical activity are associated with the poor prognosis of OC [3–7]. While the majority of patients initially respond well to chemotherapy, some patients relapse and become chemoresistant [8, 9]. Therefore, the identification of potential predictors that improve the prognosis for women diagnosed with OC may have clinical importance.

Under hypoxic conditions, tumor cells adapt by generating energy in oxygen-independent ways by inducing the expression of genes involved in tumor progression [10]. Additionally, hypoxia can increase the resistance to radiotherapy and chemotherapy and lead to the decline of cell immunity [11–13]. Moreover, hypoxic environment is significantly related to the poor prognosis in patients with OC [14]. Up to now, the detailed mechanisms by which hypoxia regulates the status of OC cells resulting in physiological changes remain unclear. Hence, exploring the effect of hypoxia on OC may offer opportunities for potential therapeutic purposes. Nowadays, cancer immunotherapy can target the cells of the immune system [15]. A detailed understanding of the interactions between cancer, hypoxia, and the immune system may be vital for the recognition of potential new immunotherapeutic strategies and targets for OC patients. In this study, we tried to use gene expression data to develop a hypoxia risk model to predict the immune microenvironment in OC patients.

## 2. Materials and Methods

### 2.1. Dataset Sources and Preprocessing

Firstly, the RNA sequencing data for gene expression (FPKM value) and clinical information were downloaded from UCSC Xena in The Cancer Genome Atlas (TCGA) dataset as a training cohort. The FPKM value was then converted to a transcript of millions per kilobase (TPM) value. Secondly, the gene expression data were downloaded from GSE17260 and GSE32062 datasets in the Gene Expression Omnibus (GEO) database as a validation set. Patient characteristics of the above three cohorts are shown in Table 1. The average value of multiple probes corresponding to the same gene was used as its expression quantity to obtain the gene expression matrix file. The “SVA” software package in R language was used for batch normalization of expression data to obtain a standardized gene expression matrix file. The detailed information of the above datasets is shown in Table 2. A list of hypoxia-related genes was obtained from Hallmark gene sets [16] of the Molecular Signatures Database. Totally, 191 genes were included, all of which responded to low oxygen levels.

### 2.2. Construction and Verification of Hypoxic Gene Signature

The analysis method was referred to the previous literature [17]. In the training cohort, the prognosis-related hypoxic genes were identified by univariate Cox regression analysis and lasso regression analysis. *p* < 0.01 was considered statistically significant. The hypoxic gene signature for predicting the prognosis of OC patients was constructed through Cox and lasso regression analyses by using the “glmnet” software package in R language. In the analysis, the lasso penalty was applied. At the same time, shrinkage and variable selection were taken into account. The composition of the final gene signature was selected to generate the risk score based on the following formula:

(1) $$\begin{array}{c} \text{risk score }=\left(\text{coefficient gene}1\;\times \text{ expression of gene}1\right)+\left(\text{coefficient gene}2\;\times \text{ expression of gene}2\right)+\cdots +\left(\text{coefficient geneN }\times \text{ expression of geneN}\right)\text{.} \end{array}$$

The cases were divided into two groups (high risk and low risk) based on the risk score median. In addition, the same formula was used to calculate the risk score in the validation set.

### 2.3. Survival Analysis

Overall survival (OS) was compared between the high hypoxia risk group and the low hypoxia risk group via Kaplan–Meier analysis. The multivariate Cox regression analysis was used to determine risk score as an independent risk factor for OS in OC. The receiver operating characteristic (ROC) curve was generated to validate the accuracy of the risk model in predicting the patients' OS via the “timeROC” software package in R language.

### 2.4. Gene Set Enrichment Analysis (GSEA)

Underlying mechanisms were studied through gene set enrichment analysis (GSEA) [18] with the Java program in the TCGA dataset. The adjusted *p* value was calculated by using the method of Benjamini–Hochberg false discovery rate (FDR). The FDR value of ≤0.05 of the enriched gene set was considered to be statistically significant.

### 2.5. Estimation of Tumor Immune Microenvironment (TIME) Cell Infiltration

The single-sample gene set enrichment analysis (ssGSEA) algorithm was used to quantify the relative abundance of TIME cell infiltration in each OC sample. The gene set for marking each TIME infiltration immune cell type was obtained from the previous study [19, 20]. The enrichment scores (calculated by ssGSEA) were used to represent the relative abundance of each TIME infiltrating cell in each sample. The immune score, matrix score, purity of tumor, and ESTIMATE score were calculated [21]. The violin plot and boxplot were used to compare the levels of immune cell infiltration and immune score between the high and low hypoxia risk groups.

### 2.6. Expression Analysis of Genes That Negatively Regulated the Cancer Immune Cycle

Cancer immune cycle describes a cycle of processes involved in the eradication of cancer by the immune system [22]. In this study, the gene signature was downloaded from the Tracking Tumor Immunophenotype website [23] to study the gene expression that negatively regulated the cancer immune cycle between the high and low hypoxia risk groups.

### 2.7. Analysis of Immune Checkpoint and Somatic Mutation

To further clarify the potential association between TIME hypoxia and clinical immunotherapy, the expression of 6 immune checkpoints including PDCD1 (PD1), CD274 (PDL1), PDCD1LG2 (PDL2), CTLA4, CD80, and CD86 were analyzed in the high hypoxia risk group and low hypoxia risk group. In addition, somatic mutation analysis was performed to identify mutation status in the high and low hypoxia risk groups. The somatic mutation data of OC patients were downloaded from the TCGA dataset. The numbers of variant types and classifications were visualized with Oncoplot.

### 2.8. In Vitro Expression Analysis of Hypoxic Gene Signature

In order to study the expression of hypoxic gene signature at the mRNA level, the RT-qPCR was performed in tissue samples. Totally, 5 patients with OC were enrolled. The inclusion criteria of the enrolled OC patients were as follows: (1) patients were diagnosed with OC, which was confirmed by pathological examination; (2) patients received no radiation, chemotherapy, immunotherapy, or molecular targeted therapy prior to diagnosis; (3) patients had no other malignant tumors and autoimmune diseases; and (4) age of patients ranging from 18 to 70. The exclusion criteria were as follows: (1) patients had other malignant tumors; (2) patients received preoperative adjuvant chemotherapy, radiotherapy, or targeted therapy; (3) patients had incomplete clinical data; and (4) patients had a history of cancer. Tumor tissue samples and adjacent tissue samples were collected from 5 OC patients. Tissue samples and clinical data were collected with informed consent of patients. This study was approved by the Ethics Committee of The Second Affiliated Hospital of Xi'an Jiaotong University (2021241).

Total RNA of the samples was extracted using the TRIzol® reagent. Then, RT-qPCR was performed in an ABI 7300 RT-qPCR system with SuperReal PreMix Plus. Relative gene expressions were analyzed by the 2^-△△ct^ method and represented as fold change (compared with healthy control). Fold change >1 and fold change <1 represented upregulation and downregulation, respectively. GAPDH and ACTB were used for internal reference.

### 2.9. Statistical Analysis

All statistics were performed using the R software. Wilcoxon test was used to identify differentially expressed genes and infiltrating immune cells. In addition, the Wilcoxon test was used to screen for differentially expressed infiltrating immune cells and to analyze statistical differences in the expression of risk scores in different clinical features. Kaplan–Meier curves were plotted, and a log-rank test was used to check the significant difference in OS between the high and low hypoxia risk groups. The *t*-test was used to test the significant difference in hypoxic gene expression between the high and low hypoxia risk groups. The value of *p* less than 0.05 was set as statistically significant.

## 3. Results

### 3.1. Construction of Hypoxic Gene Signature Prognostic Model in OC

**Figure 1:**
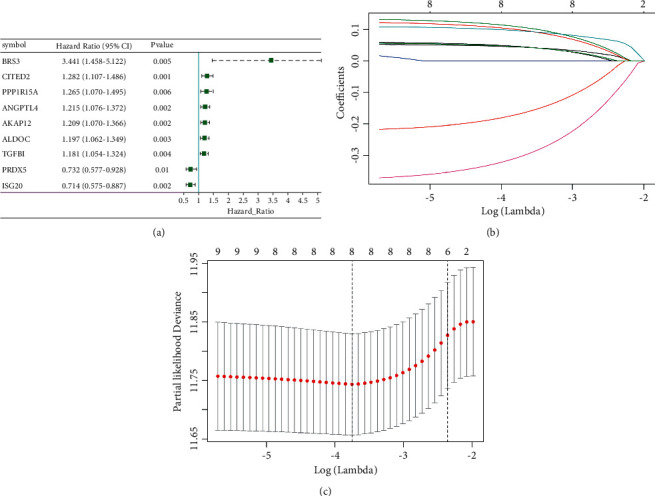
Construction of hypoxic gene signature prognostic model in OC. (a) The forest map of univariate Cox regression analysis results of hypoxia gene. (b) The coefficient profile plot. (c) Optimal parameter (lambda) selection in the lasso model used tenfold cross-validation via minimum criteria.

The prognostic role of 191 hypoxic genes in OC patients was investigated. Based on the univariate Cox regression analysis, 9 hypoxia-related genes were significantly related to patients' OS (Figure 1(a)). In the lasso and Cox regression analyses, 8 hypoxia-related genes were chosen to build the predictive model consisting of A-kinase anchoring protein 12 (AKAP12), aldolase, fructose-bisphosphate C (ALDOC), angiopoietin-like 4 (ANGPTL4), Cbp/p300 interacting transactivator with Glu/Asp-rich carboxy-terminal domain 2 (CITED2), interferon-stimulated exonuclease gene 20 (ISG20), protein phosphatase 1 regulatory subunit 15A (PPP1R15A), peroxiredoxin 5 (PRDX5), and transforming growth factor beta-induced protein (TGFBI) (Figures 1(b) and 1(c)).

### 3.2. Prognostic Value of the Hypoxia Risk Signature in OC

**Figure 2:**
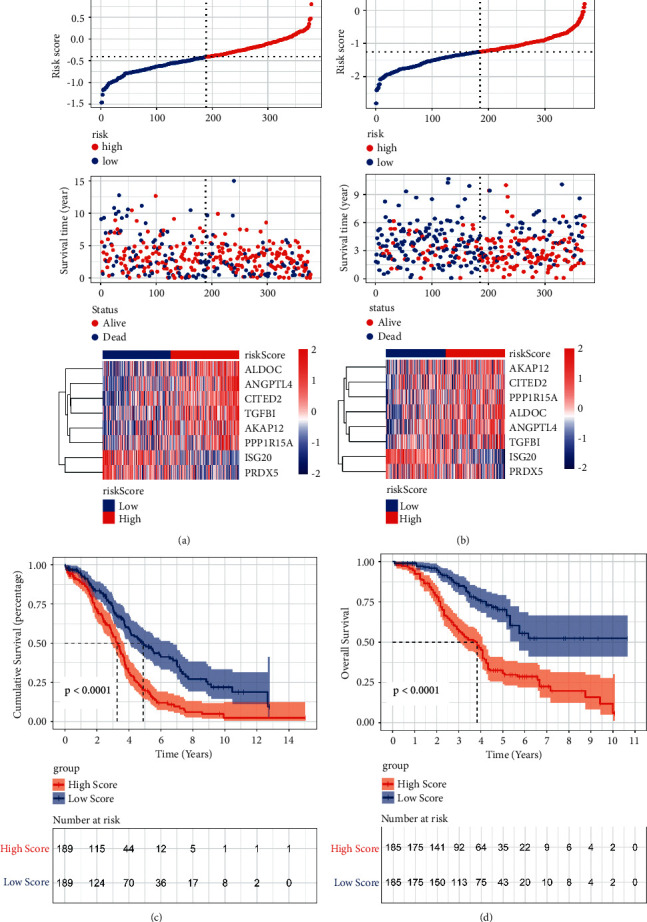
Construction and validation of prognostic hypoxia risk signature in OC. (a) Distribution of risk score, OS, and OS status and heatmap of the 8 prognostic hypoxia risk gene signature in the training cohort; (b) distribution of risk score, OS, and OS status and heatmap of the 8 prognostic hypoxia risk gene signature in the validation cohort; (c) Kaplan–Meier curves of OS for OC patients based on the risk score in the training cohort; (d) Kaplan–Meier curves of OS for OC patients based on the risk score in the validation cohort.

The risk scores of the training and validation cohorts were calculated using the coefficients obtained by the lasso algorithm. Patients were divided into high and low hypoxia risk groups. The distribution of the risk scores, OS, OS status, and expression profiles of the 8 hypoxic gene signature in the training cohort and validation cohort is displayed in Figures 2(a) and 2(b). Heat map results showed that 6 hypoxic genes, including AKAP12, ALDOC, ANGPTL4, CITED2, PPP1R15A, and TGFBI, were highly expressed in the high hypoxia risk group, which indicated that patients in the group tended to develop hypoxic microenvironments. The mortality was significantly higher in the high hypoxia risk group. The OS of the high hypoxia risk group was shorter in the training and validation cohorts (Figures 2(c) and 2(d)).

### 3.3. Strong Power of Hypoxia Risk Signature for Prognosis Assessment in OC

**Figure 3:**
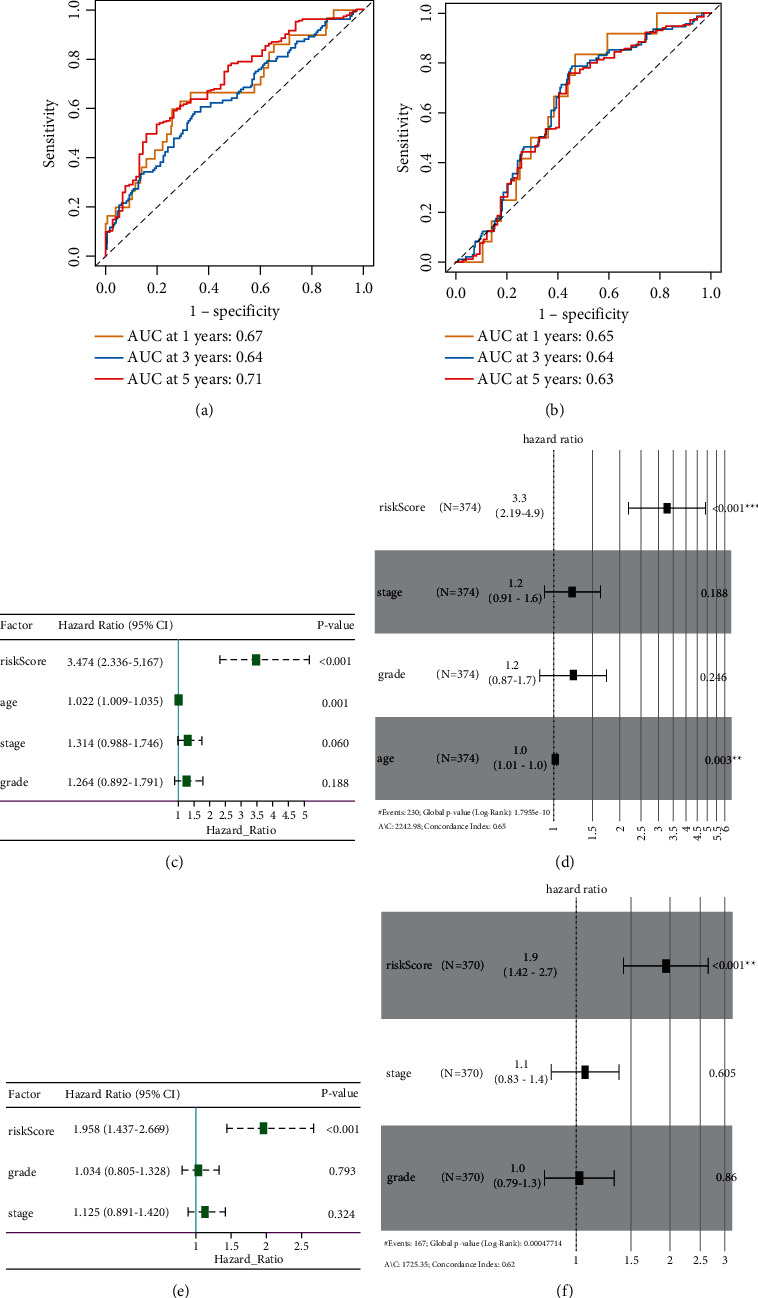
Prognostic value of the hypoxia risk signature in OC. (a, b) ROC curves showing the predictive efficiency of the hypoxia risk signature; (c–f) univariate and multivariate Cox analyses of the hypoxia signature.

**Figure 4:**
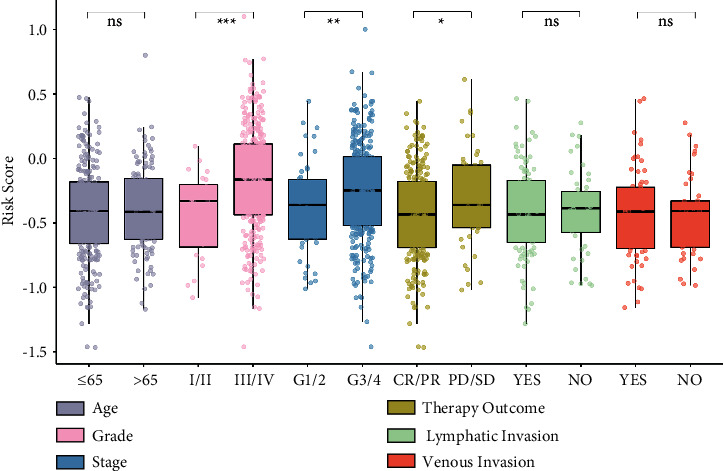
The relationship between the hypoxia signature and clinical parameters. ^*∗*^*p* < 0.05; ^*∗∗*^*p* < 0.01; ^*∗∗∗*^*p* < 0.001.

To evaluate the predictive efficiency of the hypoxia risk signature in the 1-, 3-, and 5-year survival rates, the ROC curve was performed. The AUC was 0.67 at 1 year, 0.64 at 3 years, and 0.71 at 5 years, respectively, indicating a high predictive value (Figure 3(a)). This was further validated in the validation cohort (Figure 3(b)). This indicated that patients with high risk score could develop hypoxia tumor microenvironment. The univariate analysis suggested that high hypoxia risk score was significantly associated with poor OS (Figure 3(c)). Multivariate analysis showed that high hypoxia risk score was significantly related to poorer OS of OC patients (Figure 3(d)). These were validated in the validation cohort (Figures 3(e) and 3(f)). In addition, the relationship between the hypoxia signature and clinical parameters (such as age, grade, stage, therapy outcome, lymphatic invasion, and venous invasion) is shown in Figure 4. The risk score for grade III/IV was significantly higher than that for grade I/II. The risk score of G3/4 was significantly higher than that of G1/2. In therapy outcome, the risk score of PD/SD was significantly higher than that of CR/PR.

### 3.4. Identification of Hypoxia-Related Signaling Pathways in OC

**Figure 5:**
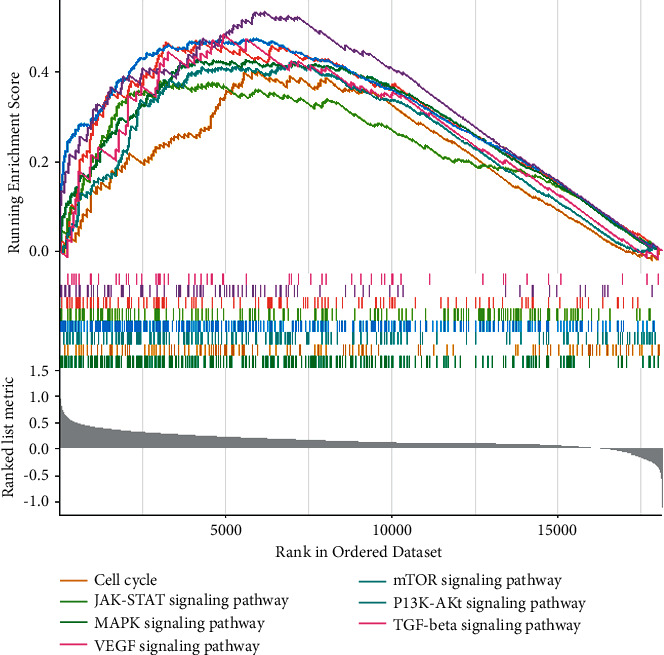
Enrichment plots of activated signaling pathways in the high hypoxia risk group.

GSEA results showed that processes associated with stimulating tumor proliferation and antiapoptosis were significantly enriched in the high hypoxia risk group (Figure 5), including cell cycle, MAPK signaling pathway, mTOR signaling pathway, PI3K-Akt signaling pathway, VEGF signaling pathway, and AMPK signaling pathway.

### 3.5. Immunity Analysis between High and Low Hypoxia Risk Groups in OC

**Figure 6:**
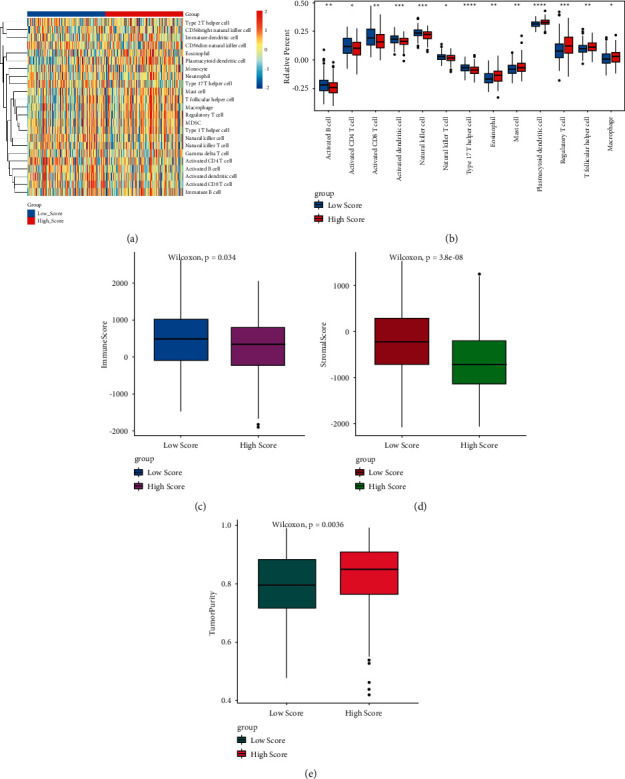
Immunity analysis between the high and low hypoxia risk groups. (a) All the immune cells; (b) differential immune cells; (c) immune score; (d) matrix score; (e) tumor purity.

The ability to assess hypoxia risk signals in the immune microenvironment was investigated through ssGSEA. Patients at high risk of hypoxia had significantly higher percentages of cells (such as plasmacytoid dendritic cell and regulatory T cell) and significantly lower percentages of activated dendritic cell, type 17 T helper cell, and natural killer cell (Figures 6(a), 6(b)). This suggested that immunosuppressive cells may drive the immunosuppressive microenvironment. The results of the ESTIMATE algorithm also confirmed that the immune score (Figure 6(c)) and matrix score (Figure 6(d)) were significantly lower in patients with high hypoxia. The tumor purity was significantly higher in patients with low hypoxia (Figure 6(e)).

### 3.6. A High Risk Score Indicates the Immunosuppressive Microenvironment in OC

**Figure 7:**
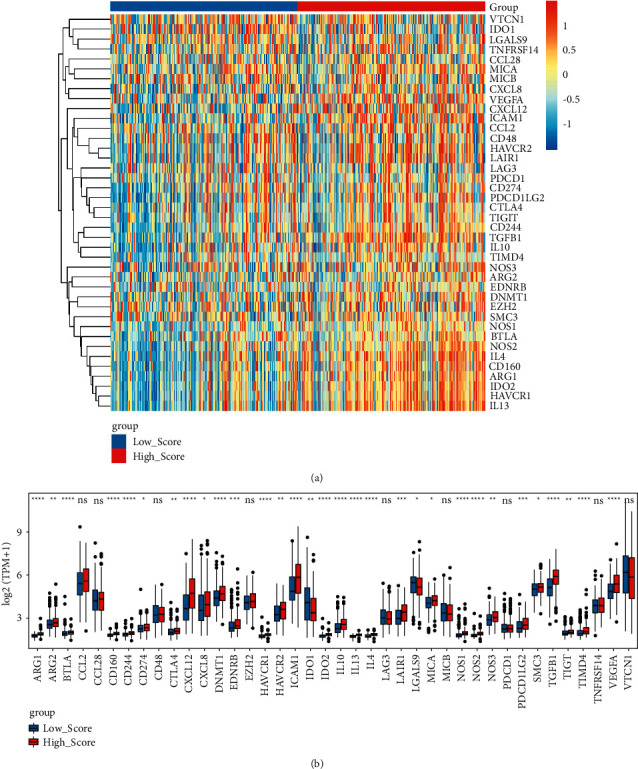
Expression of genes that negatively regulated cancer immune cycle in the high and low hypoxia risk groups. (a) Heat maps of genes that negatively regulated cancer immune cycle in the high and low hypoxia risk groups; (b) boxplots of genes that negatively regulated cancer immune cycle in the high and low hypoxia risk groups.

The gene signature that negatively regulated cancer immune cycle was downloaded from the website “Tracking Tumor Immunophenotype.” The results showed that most of these genes, such as arginase 1 (ARG1), CD 160 molecule (CD160), CD 244 molecule (CD244), C-X-C motif chemokine ligand 12 (CXCL12), DNA methyltransferase 1 (DNMT1), and hepatitis A virus cellular receptor 1 (HAVCR1), were upregulated in the high hypoxia risk group (Figure 7).

### 3.7. Analysis of Expression Patterns and Mutation Types of Immune Checkpoints in OC

**Figure 8:**
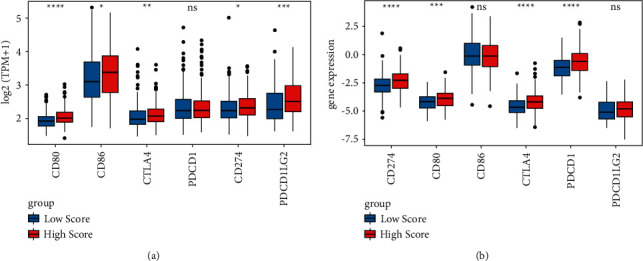
Expression of immune checkpoints in the high and low hypoxia risk groups. (a) In the training cohort; (b) in the validation cohort.

**Figure 9:**
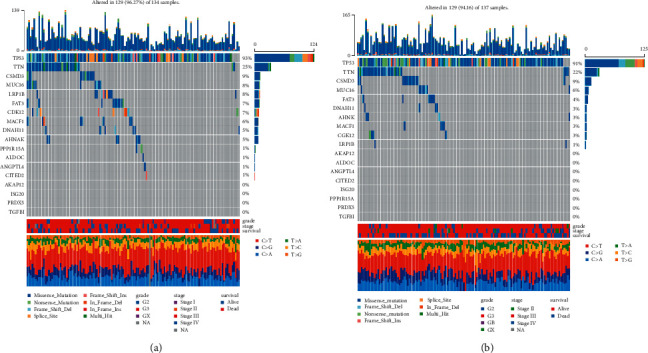
The waterfall plot of tumor somatic mutation established by mutations. (a) High hypoxia risk group; (b) low hypoxia risk group. Each column represents individual patients. The upper barplot shows tumor mutation burden, and the number on the right indicates the mutation frequency in each gene. The right barplot shows the proportion of each variant type.

The expression of immune checkpoints was investigated in the high and low hypoxia risk groups. The results showed that the expression of most immune checkpoints, such as CD80 molecule (CD80), cytotoxic T-lymphocyte-associated protein 4 (CTLA4), and cd274 molecule (CD274), were significantly higher in the high hypoxia risk group (Figure 8(a)), which was validated in the validation cohort (Figure 8(b)). These results suggested that the immune microenvironment in the high hypoxia risk group was suppressed by upregulating immunosuppressive cytokines and immune checkpoints. In addition, the difference in gene mutation frequency was analyzed between the high and low hypoxia risk groups (Figure 9). The mutation frequency of ALDOC, ANGPTL4, and PPP1R15A was slightly higher in the high hypoxia risk group (1%) compared with that in the low hypoxia risk group (did not show any mutations). This suggested that mutation of these genes may be associated with hypoxia.

### 3.8. RT-qPCR

**Figure 10:**
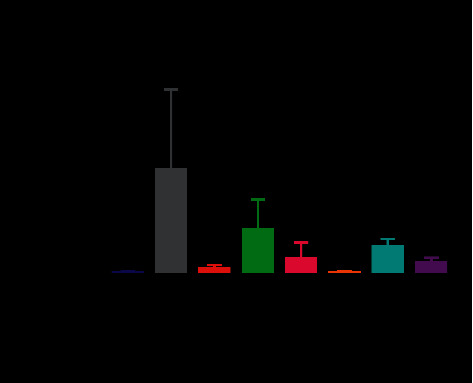
Expression of detection of hypoxic gene signature by RT-qPCR. ^*∗*^*p* < 0.05; ^*∗∗*^*p* < 0.01.

The tumor tissues from 5 patients with OC were used to test the expression of hypoxic gene signature. The clinical information of these patients is shown in Table 3. In addition, the sequence of the primers used for the RT-qPCR is listed in Table 4. The results showed that ALDOC, CITED2, ISG20, and PRDX5 were significantly upregulated, AKAP12, ANGPTL4, and PPP1R15A were remarkably downregulated, and TGFBI was downregulated without significant difference (Figure 10). This suggested that the expression level of these genes in tumor tissue was different from that under the hypoxic tumor environment.

## 4. Discussion

In the present study, we developed a risk model consisted of 8 hypoxia-associated genes in OC. Among which, AKAP12, ALDOC, ANGPTL4, CITED2, PPP1R15A, and TGFBI were highly expressed, whereas ISG20 and PRDX5 were lowly expressed in the high hypoxia risk patients. The upregulation of AKAP12 is related to poor survival of colorectal cancer [24]. It is suggested that AKAP12 plays a crucial role in ovarian granulosa cells and is involved in invasion and metastasis in OC [25–27]. Elevated AKAP12 transcript expression is related to poor survival in patients with ovarian cancer and high-grade serous ovarian carcinomas [28–30]. ALDOC, a glycolytic enzyme, is known to be upregulated by hypoxia. In OC, increased mRNA expression of ALDOC fuels the tricarboxylic acid cycle and promotes sustained mitochondrial respiration [31]. ANGPTL4, an immune gene, has important functions in lipid and glucose metabolism [32, 33]. ANGPTL4 is activated by hypoxia-inducible factor-1*α* (HIF-1*α*) and confers protection against hypoxia-induced apoptosis [34]. It is demonstrated that ANGPTL4 is overexpressed in OC and related to shorter relapse-free survival times in serous OC [35–37]. In addition, ANGPTL4 is significantly related to the poor prognosis of patients with non-small-cell lung cancer, lung adenocarcinoma, and cervical cancer [38–40]. CITED2 is significantly enriched in regulatory T cells and granulosa cells [41, 42]. It is reported that CITED2 is associated with primary ovarian insufficiency [43]. Upregulation of CITED2 is related to shorter recurrence times in serous ovarian tumors [44]. It is shown that TGFBI functions as a tumor promoter in OC [31]. It is reported that TGFBI is related to an extracellular matrix signature and is implicated in poor prognosis and drug resistance in OC [45–48]. Overexpression of TGFBI is related to poor prognosis in cervical cancer [49]. ISG20, an immune-related gene, is downregulated in small atretic follicles with respect to healthy follicles [50]. The expression level of ISG20 is lower in tumor tissues of OC and is associated with the prognosis of OC [51, 52]. PRDX5 can be used to predict poor progression-free survival for OC patients [53]. Our results suggested that the above 8 hypoxia-associated gene risk model could be used as an independent prognostic factor for OC patients, which may represent a convenient detection in clinic.

In OC, regulatory T cells play roles in reducing survival [54]. In the TIME, the presence of immature plasmacytoid dendritic cells is associated with the poor clinical outcome in patients with breast cancer and epithelial OC [55, 56], which indicates that plasmacytoid dendritic cells could play roles in establishing the TIME mediated by forkhead box P3 (Foxp3)^+^ regulatory T cells. Activated dendritic cells can boost the patients' immune system to fight against cancer [57, 58]. In this study, we found that patients with high hypoxia risk had significantly higher proportions of regulatory T-cell and plasmacytoid dendritic cell phenotypes. Besides, activated dendritic cells were decreased in the high hypoxia group. In addition, the immune score and matrix score were significantly lower in patients with high hypoxia. Our results showed that the hypoxia risk model could predict the immune microenvironment in OC.

Identification of immunosuppressive factors produced within the TIME and the ability to target these factors can enhance antitumor immune responses. The expression of ARG1 in immune cells is a potent suppressor of antitumor T cells [59]. CD160 is significantly upregulated in OC [60]. CD244 is an exhaustion marker of T cells in OC [61]. Hypoxia induces the expression of CXCL12 in primary human ovarian tumor cells [62]. It is suggested that CXCL12 is an independent predictor of poor survival in OC [63]. Overexpression of DNMT1 contributes to gene promoter hypermethylation and is associated with the malignant potential and poor prognosis of breast cancer [64, 65]. HAVCR1 serves as the marker for ovarian clear cell carcinoma susceptibility [66]. In the present study, the above immunosuppressive cytokines were upregulated in the high hypoxia risk group, which further promoted immunosuppression in OC.

The immune checkpoint inhibitor-based antibody has improved survival for patients with various cancer types, such as lung cancer, malignant melanoma, and bladder cancer [67]. High levels of CD80 contribute to the maintenance of tolerance and immunosuppression in epithelial OC [68]. The OC microenvironment can induce migration of CTLA4^+^ regulatory T cells via C-C motif chemokine ligand 22 (CCL22) and C-C motif chemokine receptor 4 (CCR4) [54, 69]. CTLA4 immunotherapy has shown an optimistic antitumor effect in OC [70]. PD-L1 interacts with the corresponding receptor, inhibits the antitumor activity of immune cells, and allows cancer cells to escape immune surveillance [71]. Drug-resistant OC cells exhibit repression of immune-stimulatory molecules, with concomitant augmented expression of CD274 [72]. In this study, we found that the above immune checkpoints including CD80, CTLA4, and CD274 were all upregulated in the high hypoxia risk group, which indicated these immune checkpoints play an important immunosuppression action in OC.

Based on functional analysis, we found that gene sets associated with the high hypoxia risk group were involved in signaling pathways of cell cycle, MAPK, mTOR, PI3K-Akt, JAK-STAT, VEGF, TGF-beta, and AMPK. Genes involved in cell cycle play a key role in OC development and prognosis [73]. In in vitro conditions, induction of CD8^+^ regulatory T cell is critically mediated by the activation of p38MAPK in OC immunotherapy [74]. The mTOR pathway is found to be activated in about half of patients with high-grade serous OC [75]. The PI3K-Akt signaling pathway plays crucial roles in the process of mesenchymal stem cells induced by hypoxia [76]. Activation of the PI3K/Akt/mTOR pathways is found in OC [77]. VEGF, an important angiogenic factor in advanced OC, is related to tumor aggression and the poor prognosis of OC [78, 79]. AMPK protects living cells from hypoxia, which results in elevations in the cellular AMP/ATP ratio [80–82]. It is found that AMPK subunits are generally upregulated in OC [83]. Our results indicated that the above signaling pathways may play important roles in the low oxygen environment of OC.

CA: carcinoma antigen; HE4: rabbit anti-human HE4 antibody.

## 5. Conclusions

We developed and validated a risk model based on 8 hypoxia-associated genes, which could serve as an independent prognostic factor for OC patients and reflect the overall intensity of the immune response in the OC microenvironment. Our study may offer a novel understanding of how hypoxia status affects prognosis and the TIME in OC and benefits future hypoxia-targeted therapies. However, there are limitations to our study. More molecular investigation in the experimental model is further needed.

## Figures and Tables

**Table 1:** Patient characteristics of three cohorts.

	TCGA	GSE17260	GSE32062
Number of patients	378	110	260
Age (median, range)	59 (30–87)	NA	NA
Grade (%)			
Grade 1	1 (0.26%)	26 (23.64%)	NA
Grade 2	45 (11.90%)	41 (37.27%)	131 (50.38%)
Grade 3	321 (84.92%)	43 (39.09%)	129 (49.62%)
Grade 4	1 (0.26%)	NA	NA
Unknown	10 (2.65%)	NA	NA
Stage (%)			
I	1 (0.26%)	NA	NA
II	23 (6.08%)	NA	NA
III	294 (77.78%)	93 (84.55%)	204 (78.46%)
IV	57 (15.08%)	17 (15.45%)	56 (21.54%)
Unknown	3 (0.79%)	NA	NA
Lymphatic invasion			
Yes	101 (26.72%)	NA	NA
No	48 (12.70%)	NA	NA
Unknown	229 (60.58%)	NA	NA
Venous invasion			
Yes	64 (16.93%)	NA	NA
No	41 (10.85%)	NA	NA
Unknown	273 (72.22%)	NA	NA
OS days (median)	1024	915	1245

OS: overall survival; NA: not applicable.

**Table 2:** Basic information of all datasets.

Accession number	Platform	Number of patients	Survival data
TCGA	Illumina RNA-seq	OC = 378	OS
GSE17260	Agilent-014850 Whole Human Genome Microarray 4 × 44K G4112F	OC = 110	OS
GSE32062	Agilent-014850 Whole Human Genome Microarray 4 × 44K G4112F	OC = 260	OS

OC: ovarian cancer; OS: overall survival.

**Table 3:** The clinical information of enrolled patients in intro validation.

Number	Age	Height (cm)	Weight (kg)	Tumor size (cm)
1	58	156	54	1
2	62	157	59	10
3	70	159	50	8
4	73	168	46	20
5	56	165	53	10

**Table 4:** The sequence of the primers used for the RT-qPCR.

Primer name	Primer sequences (5′ to 3′)	Size (bp)
GAPDH-F (internal reference)	CTGGGCTACACTGAGCACC	101
GAPDH-R (internal reference)	AAGTGGTCGTTGAGGGCAATG	
ACTB-F (internal reference)	GATCAAGATCATTGCTCCTCCT	108
ACTB-R (internal reference)	TACTCCTGCTTGCTGATCCA	
AKAP12-F	AGAGGTTGCCTCCGAGAAACT	185
AKAP12-R	CAAACACTTCTGTCGCCAACG	
ALDOC-F	CGCAGCCTCATTTACCAGA	165
ALDOC-R	GCTCCTTCCAAGGCTTCAG	
ANGPTL4-F	GGCTCAGTGGACTTCAACCG	103
ANGPTL4-R	CCGTGATGCTATGCACCTTCT	
CITED2-F	ACAAACCAGCACTTCCGAGAT	212
CITED2-R	TCTATGACATTGGGCGGCAG	
ISG20-F	CGACAAGTTCATCCGGCCT	176
ISG20-R	GCCACAACAGCCTGTCAGT	
PPP1R15A-F	ATGATGGCATGTATGGTGAGC	120
PPP1R15A-R	AACCTTGCAGTGTCCTTATCAG	
PRDX5-F	GCAAGACGGTGCAGTGAAG	98
PRDX5-R	ATGGCATCTCCCACCTTGATT	
TGFBI-F	CTTCGCCCCTAGCAACGAG	155
TGFBI-R	TGAGGGTCATGCCGTGTTTC	

## Data Availability

All data are available in the article.
